# Evaluation of the effect of hemodynamic factors on retinal microcirculation by using 3D confocal image-based computational fluid dynamics

**DOI:** 10.3389/fbioe.2024.1489172

**Published:** 2024-11-27

**Authors:** Chi Wei Ong, Bingyao Tan, Shaista Hussain, Thanadet Chuangsuwanich, Fabian Albert Braeu, Fangsen Cui

**Affiliations:** ^1^ School of Chemistry, Chemical Engineering and Biotechnology, Nanyang Technological University, Singapore, Singapore; ^2^ Department of Ocular Imaging, Singapore Eye Research Institute, Singapore, Singapore; ^3^ SERI NTU Advanced Ocular Engineering (STANCE) Program, Nanyang Technological University, Singapore, Singapore; ^4^ Institute of High Performance Computing (IHPC), Agency for Science, Technology and Research (A*STAR), Singapore, Singapore; ^5^ Campus for Research Excellence and Technological Enterprise (CREATE), Singapore, Singapore

**Keywords:** hemodynamic, microcirculation, CFD, retinal, confocal

## Abstract

**Purpose:**

To investigate local hemodynamic changes resulting from elevated intraocular pressure (IOP) in different vasculature networks using a computational fluid dynamics model based on 3D reconstructed confocal microscopic images.

**Methods:**

Three-dimensional rat retinal vasculature was reconstructed from confocal microscopy images using a 3D U-Net-based labeling technique, followed by manual correction. We conducted a computational fluid dynamics (CFD) analysis on different retinal vasculature networks derived from a single rat. Various venule and arteriole pressures were applied to mimic the effects of elevated intraocular pressure (IOP), a major glaucoma risk factor. An increase in IOP typically correlates with a decrease in venous pressure. We also varied the percentage of capillary dropout, simulating the loss of blood vessels within the capillary network, by reducing the volume of the normal capillary network by 10%, 30%, and 50%. Based on the output of the CFD analysis, we calculated velocity, wall shear stress (WSS), and pressure gradient for different vasculature densities.

**Results:**

Arteriolar pressure, venular pressure, and capillary dropout appear to be important factors influencing wall shear stress in the rat capillary network. Our study revealed that the pressure gradient between arterioles and venules strongly affects the local wall shear stress distribution across the 3D retinal vasculature. Specifically, under a pressure gradient of 3,250 Pa, the wall shear stress was found to vary between 0 and 20 Pa, with the highest shear stress observed in the region of the superficial layer. Additionally, capillary dropout led to a 25% increase or decrease in wall shear stress in affected areas.

**Conclusion:**

The hemodynamic differences under various arteriole and venule pressures, along with different capillary dropout conditions, could help explain the development of various optic disorders, such as glaucoma, diabetic retinopathy, and retinal vein occlusion.

## 1 Introduction

Glaucoma is a multifactorial disease and a leading cause of irreversible blindness, driven by complex interactions among intraocular pressure (IOP), vascular dysregulation, and biomechanical stress within the eye. While numerous models have been developed to explore these mechanisms, many focus on isolated aspects of the disease or oversimplify the intricate three-dimensional structures of the eye. For example, Arciero et al. (2013) focused on the vascular regulation of ocular blood flow, providing important insights into the vascular contributions to glaucoma progression. Similarly, Albright et al. (2023) examined how both increased intraocular pressure (IOP) and impaired flow regulation mechanisms can lead to decreased retinal tissue oxygenation. Guidoboni et al. (2014) introduced a mathematical model to clarify the relationship between IOP, blood pressure (BP), and retinal blood flow autoregulation (AR), highlighting their clinical relevance in glaucoma.

Despite these advancements, many existing models rely on simplified geometries or primarily on human-based data, limiting their applicability in preclinical research, where animal models like rats play a crucial role. The use of simplified anatomical structures constrains these models’ ability to fully represent the complex three-dimensional interactions within the eye. Moreover, while these models offer valuable insights into human glaucoma, they are less suited for studying disease mechanisms in animal models, which are essential for preclinical testing and translational research.

Wall shear stress may play a key role in the pathogenesis of ocular disease, where both mechanical and vascular factors have been implicated; however, a clear connection between these biomechanical factors remains elusive. Bernabeu et al. (2014) used single confocal images to study the complex behavior of hemodynamic forces in a mouse retinal model of angiogenesis. Their findings showed that the changes in velocity and the wall shear stress (WSS) gradient can affect the alteration of capillary network density, potentially influencing vessel regression. Past studies have shown that vasoconstriction and dilation are mechanosensitive problems, and disrupted fluid wall shear stress can influence the capillary remodeling process (Santamaría et al., 2020). Long exposure to disturbed flow regions may upregulate pro-inflammatory genes and proliferation, while short exposure to varying directions of shear stress may trigger the morphological changes in endothelial cells that can be relevant to capillary remodeling (Wang et al., 2007; Chiu and Chien, 2011; Mack et al., 2017; Santamaría et al., 2020). Therefore, a detailed investigation of the mechanism behind hemodynamic changes in 3D retinal capillary networks and their propensity to present in specific sections of capillary networks could enhance our understanding of the pathophysiology of capillary networks in glaucoma.

In response to these challenges, we present a novel three-dimensional computational model of glaucoma based on rat anatomy combined with computational fluid dynamics (CFD). This model represents a significant advancement over previous approaches by incorporating the detailed geometry of the rat eye, which is widely used in experimental glaucoma research. The anatomical precision of our 3D rat model allows for more realistic simulations, making it a valuable tool for preclinical research. Through this work, we aim to bridge the gap between preclinical animal research and computational modeling in glaucoma, providing a more accurate and applicable framework for studying disease mechanisms and evaluating potential therapeutic strategies.

## 2 Methodology

### 2.1 Data acquisition of 3D rat retinal confocal images

A male Brown Norway rat (∼300 g) was used in this study. The rat was bred and treated in accordance with the ARVO Statement for the Use of Animals in Ophthalmic and Vision Research. This study was approved by the SingHealth Institutional Animal Care and Use Committee (SHS/2017/354). The detailed protocol for the experiment can be found in our previous article (Chong et al., 2020). Volumetric scans were collected over a 2.3 mm × 2.3 mm × 0.06 mm region centered at the optic nerve head, with each volume scan consisting of 75 slices.

### 2.2 Segmentation process of rat retinal images

**FIGURE 1:**
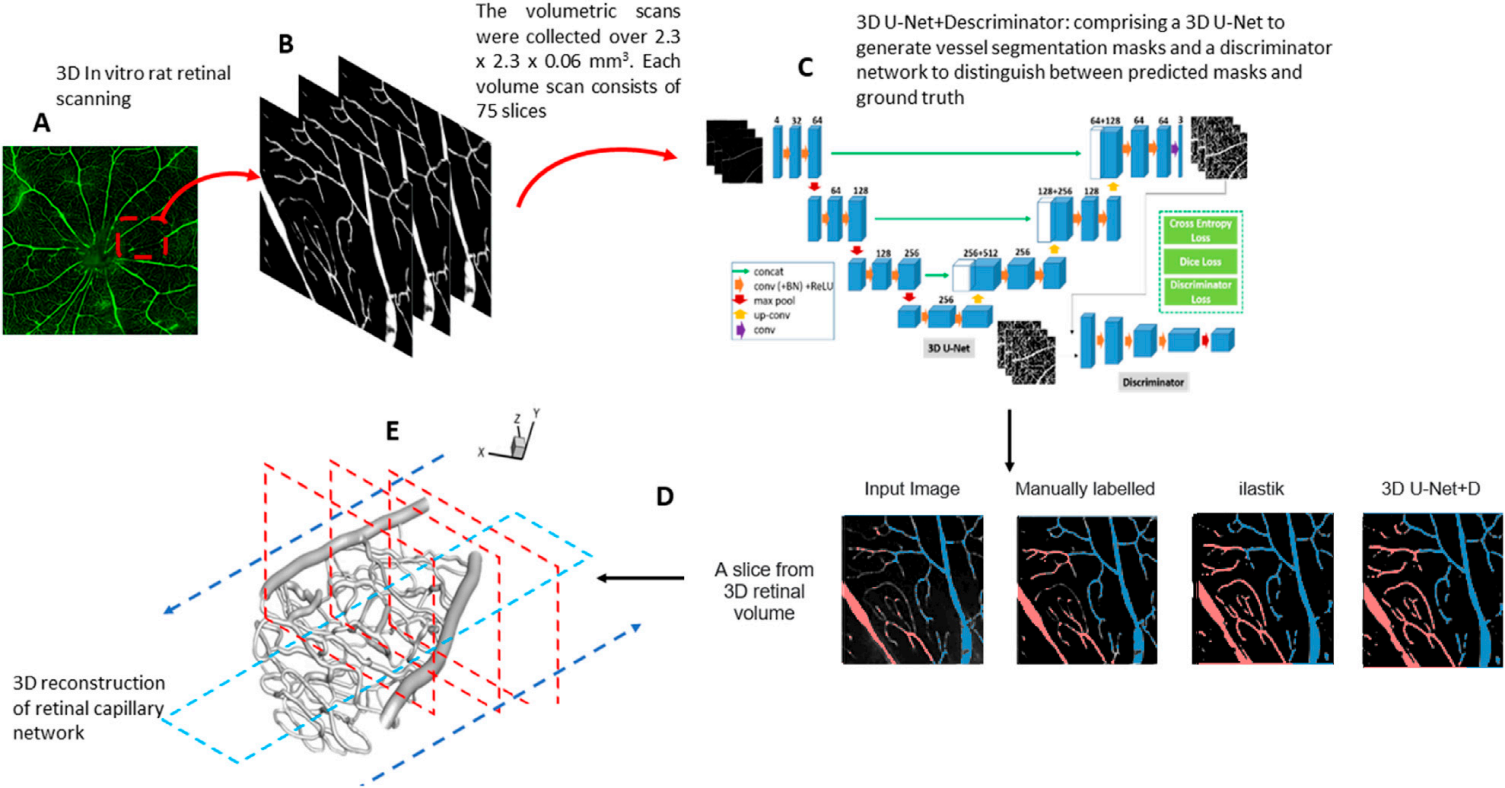
**(A)** Confocal fluorescent image of a whole rat retina. **(B)** The volumetric scans were collected over 2.3 mm × 2.3 mm × 0.06 mm. Each volume scan consists of 75 slices. **(C)** An overview of the 3D GAN model used for segmentation. **(D)** Comparison between 3D GAN and the semi-supervised random forest-based method (ilastik). We label the veins (blue) and arteries (red). **(E)** 3D reconstruction of the retinal capillary network. The arrow indicates the workflow direction.

We propose a novel method to segment small capillaries and large arterioles/venules from confocal 3D images of the rat retina, as shown in Figure 1. The segmentation process is carried out using a 3D generative adversarial network (GAN), pre-trained on synthetic 3D mouse brain images (Todorov et al., 2020). This pre-training was essential to overcome the limitations of a small labeled retinal vessel dataset, allowing the model to learn general features of vascular structures, which could then be fine-tuned for our specific retinal data.

Each confocal image volume consists of a stack of 75 individual 2D images captured at intervals to create a complete 3D reconstruction of the vascular network. Prior to segmentation, the images were preprocessed to remove noise and enhance vessel contrast using a Gaussian filter. The 3D GAN used for the segmentation includes a generator and a discriminator. The generator is based on a U-Net architecture with three down-sampling blocks, each consisting of 3D convolutions with a kernel size of 4, stride 2, and same padding, followed by instance normalization and a leaky rectified linear unit (ReLU) activation function. There are three up-sampling blocks, each of them made by 3D transpose convolutions using kernel size of 4 and stride 2, followed by instance normalization and ReLU activation. The bottleneck layer of U-Net consists of three residual blocks. The discriminator network comprises three down-sampling blocks, similar to the generator.

Data augmentation techniques, including rotation, flipping, and intensity scaling, were applied to increase the model’s robustness to variations in vessel orientation and image intensity. Both the generator and discriminator of the 3D GAN were trained using the Adam optimizer on sub-volumes of 128 × 128 × 128 voxels, with a batch size 4, over 200 epochs. Our deep learning-based, fully automated 3D retinal vessel segmentation method achieved a Dice coefficient of 0.7, which is a commonly used metric to evaluate segmentation accuracy. The Dice coefficient measures the overlap between the predicted segmentation and the ground truth, ranging from 0 (no overlap) to 1 (perfect overlap).

Following segmentation, the results were refined manually using Rhino (McNeel, 2010), a 3D modeling software, to correct any misclassified vessels or artifacts generated during the automated process. This manual correction involved identifying regions where the segmentation model over-segmented or under-segmented small vessels and adjusting these using polygonal mesh editing tools within Rhino, ensuring the integrity of the vascular network was maintained. The corrected segmentation was then validated by comparing it against ground truth annotations completed by our expert team from the Singapore Eye Research Institute.

### 2.3 Computational fluid dynamics analysis

The 3D geometry of the retinal capillary network was reconstructed using a suite of software, including Mimics 21.0 (Materialize NV, Belgium),based on the segmented images. The reconstruction was then examined and manually refined. Subsequent smoothing of the capillary network surface and volume generation were completed using ANSYS ICEM CFD meshing software 16.0 (ANSYS, Inc., United States). A tetrahedral element was chosen for the domain discretization due to the complexity of the 3D geometry. The mesh was refined to be finer near the capillary wall, where the flow field exhibited higher complexity. The total number of elements for all geometries is higher than one million to adequately resolve the blood flow. We conducted a mesh independence test, which demonstrated a less than 3% variation between different mesh sizes in the velocity profile, thereby verifying our model numerically. A detailed table quantifying these results is provided in Supplementary Table A1.

### 2.4 Numerical formulation and governing equations

The governing equations of blood flow are described by the flow continuity equation and the momentum equation, as shown in Equations 1, 2:

∇⋅u=0, (1)

$$\rho \left(\frac{\partial u}{\partial t}+u\cdot \nabla u\right)=-\nabla p+\nabla \cdot \tau ,$$ (2)

where **u** is the fluid velocity vector, and *p*, *ρ*, and τ are the pressure, the density (ρ = 1,050 kg/m^3^), and the stress tensor, respectively. The stress tensor ***τ*** is shown in Equation 3,

$$\tau =2\eta \left(\dot{\gamma }\right)D,$$ (3)

where **D** and $\dot{\gamma }$ are the rate of the deformation tensor and the shear rate, respectively. $\dot{\gamma }$ is related to the second invariant of **D**, and η is the fluid viscosity, which is a function of $\dot{\gamma }$ .

CFD simulations were performed using the finite volume approach with Ansys-FLUENT 17.2 (ANSYS, Canonsburg, PA) to determine the flow characteristics of the three-dimensional incompressible non-Newtonian fluid. In our study, we adopted the approach described by Bernabeu et al. (2014), where experimental measurements were used to derive a functional form for viscosity based on the Carreau–Yasuda (CY) model (Tan et al., 2009), as shown in Equation 4,

$$\eta \dot{\left(\gamma \right)}=\eta_{\infty }+\left(\eta_{0}-\eta_{\infty }\right){\left[1+{\left(\lambda \dot{\gamma }\right)}^{a}\right]}^{\frac{n-1}{a}},$$ (4)

where $\eta_{\infty }$ and $\eta_{0}$ are the infinite and zero shear rate viscosities, respectively, and λ is the relaxation time constant.

The CY model is particularly well-suited for modeling non-Newtonian fluids that exhibit shear-thinning behavior. The viscosity in the CY model transitions between two Newtonian regimes: at low shear rates, the fluid maintains a constant viscosity (η₀), while at high shear rates, the viscosity asymptotically approaches a different constant value (η∞). In between these regions, the model captures the shear-thinning behavior, where the viscosity decreases as the shear rate increases. This rheological behavior is typical of biological fluids such as blood, which justifies the use of the Carreau–Yasuda model in our flow simulations.

We also employed an iterative approach to calculate the flow fields. Initially, we used the Navier–Stokes equations to estimate the velocity field while applying the CY model to iteratively determine the effective viscosity at different shear rates. This iterative process ensures that the effective viscosity and flow field are updated in tandem to accurately capture the non-Newtonian behavior. The least-squares method was employed to fit the parameters of the CY model, ensuring that the viscosity–shear rate relationship was accurately represented, similar to the approach used by Bernabeu et al. (2014).

### 2.5 Boundary conditions

The pressure gradient between the arterioles and venules was used to conduct the steady-state CFD simulations, as summarized in Tables 1, 2.

**TABLE 1:** Summary of different arteriole pressures.

	Arterioles	Venules
Baseline pressure	100 mmHg	10 mmHg
Elevated arteriole pressure (1)	130 mmHg	10 mmHg
Elevated arteriole pressure (2)	150 mmHg	10 mmHg

**TABLE 2:** Summary of different venule pressures.

	Arterioles	Venules
Baseline pressure	100 mmHg	10 mmHg
Elevated venule pressure (1)	100 mmHg	30 mmHg
Elevated venule pressure (2)	100 mmHg	50 mmHg

**FIGURE 2:**
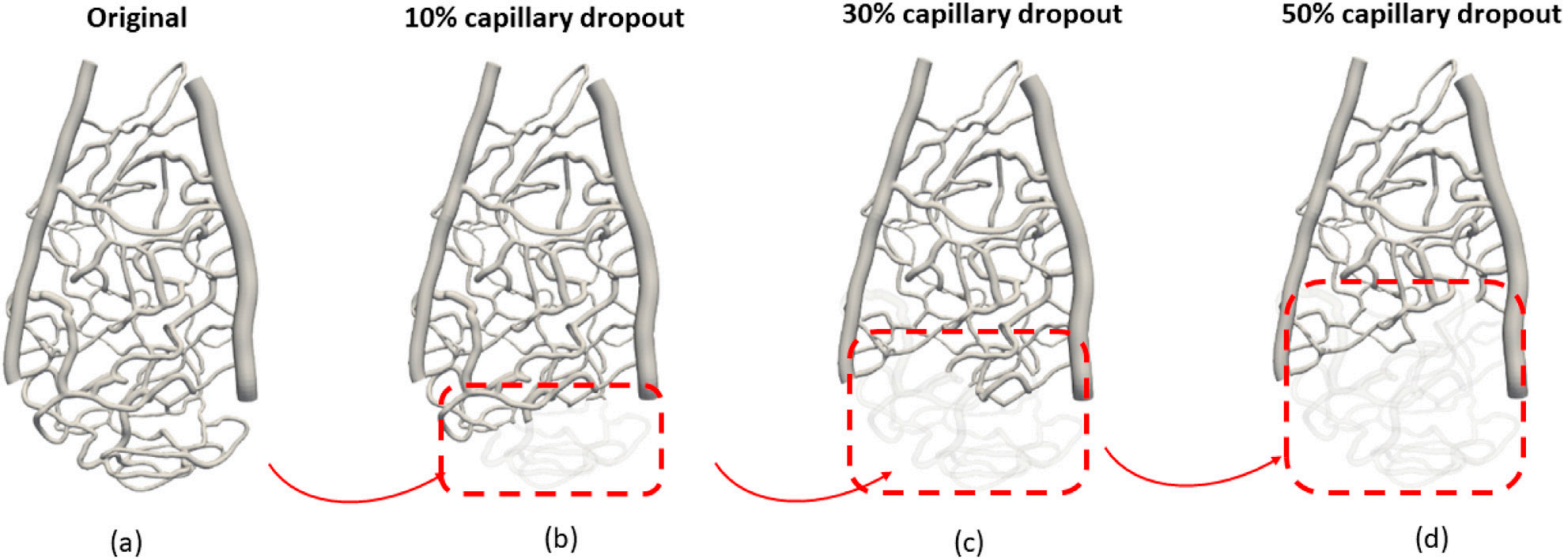
Investigation of different vascular structures to the hemodynamic distribution: **(A)** original capillary network, **(B)** 10% capillary dropout, **(C)** 30% capillary dropout, and **(D)** 50% capillary dropout.

Next, we simulated the dropout conditions shown in the red-dashed region in Figure 2, as this specific area is representative of general capillary networks, including vessels of varying sizes that significantly influence the hemodynamic pattern. By focusing on this region, we can effectively assess the impact of capillary density variations on wall shear stress and velocity profiles.

The dropout conditions were simulated at varying levels (original, 10%, 30%, and 50% volume reductions from baseline) to systematically evaluate how gradual reductions in the capillary volume affect flow dynamics. This approach allows us to capture the physiological relevance of capillary dropout and its implications for hemodynamic patterns. The selected region provides a controlled environment to observe these changes while ensuring that our findings can be extrapolated to broader vascular scenarios.

## 3 Results

### 3.1 Differences in average wall shear stress between each rat capillary region

The average wall shear stress is shown in Supplementary Figure A1. Notably, in Supplementary Figure A1, the wall shear stress at section 1, which is farther from the optic disk, was significantly higher than the wall shear stress near the optic disk (sections 2 and 3). Additionally, the average wall shear stress was markedly higher (>3 Pa) when there was more than 30% capillary dropout, compared to only a 10% capillary dropout, which resulted in only a 10% change in wall shear stress magnitude. The overall average wall shear stress across the three capillary sections was 14 ± 3.84 Pa. We also found that most regions with high wall shear stress (>15 Pa) were in the middle region between the arterioles and venules.

### 3.2 Effects of capillary dropout on wall shear stress distribution

**FIGURE 3:**
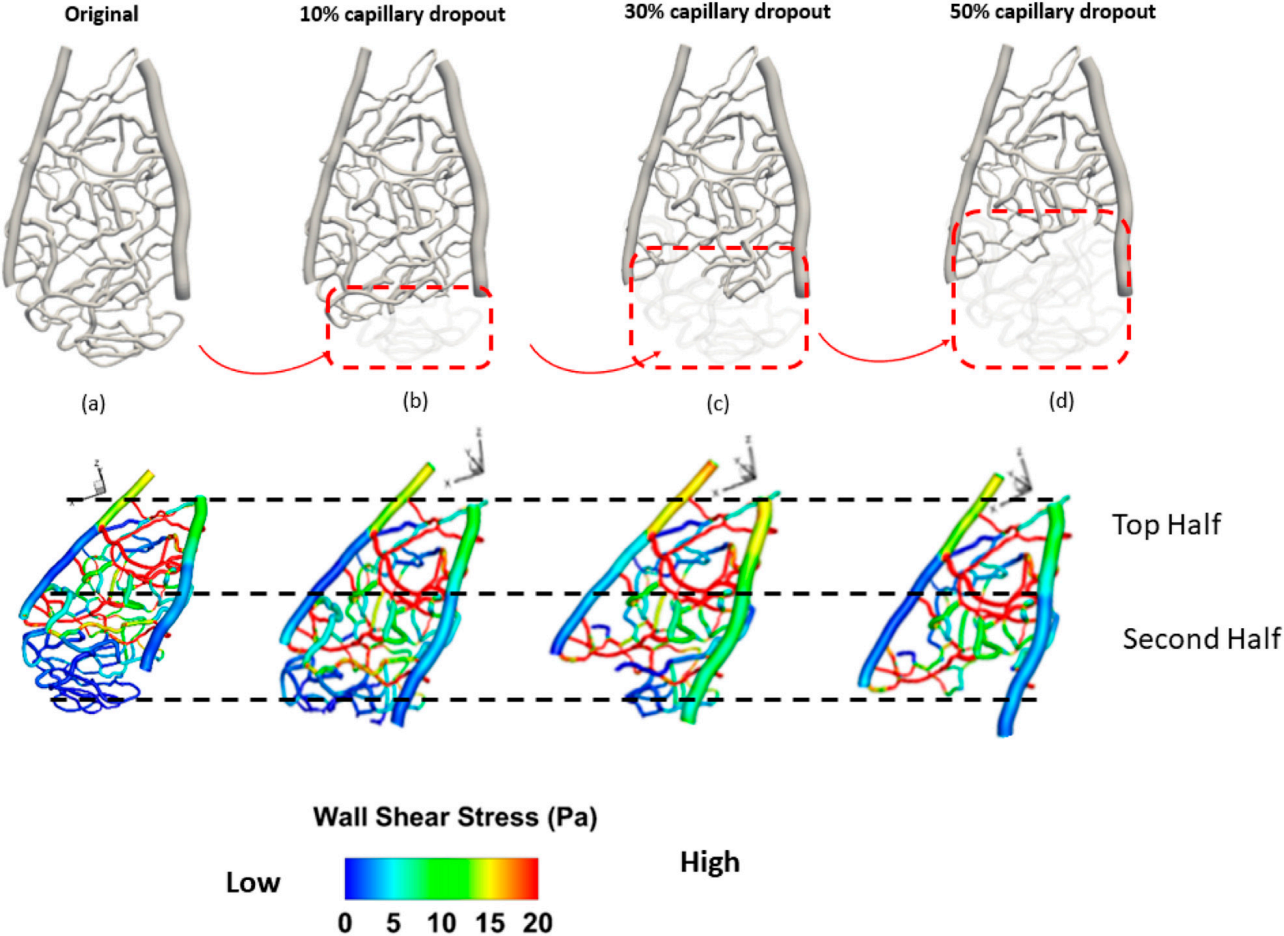
Spatial distribution of wall shear stress of a section of the rat retinal capillary network. **(A)** original capillary network, **(B)** 10% capillary dropout, **(C)** 30% capillary dropout, and **(D)** 50% capillary dropout.

**FIGURE 4:**
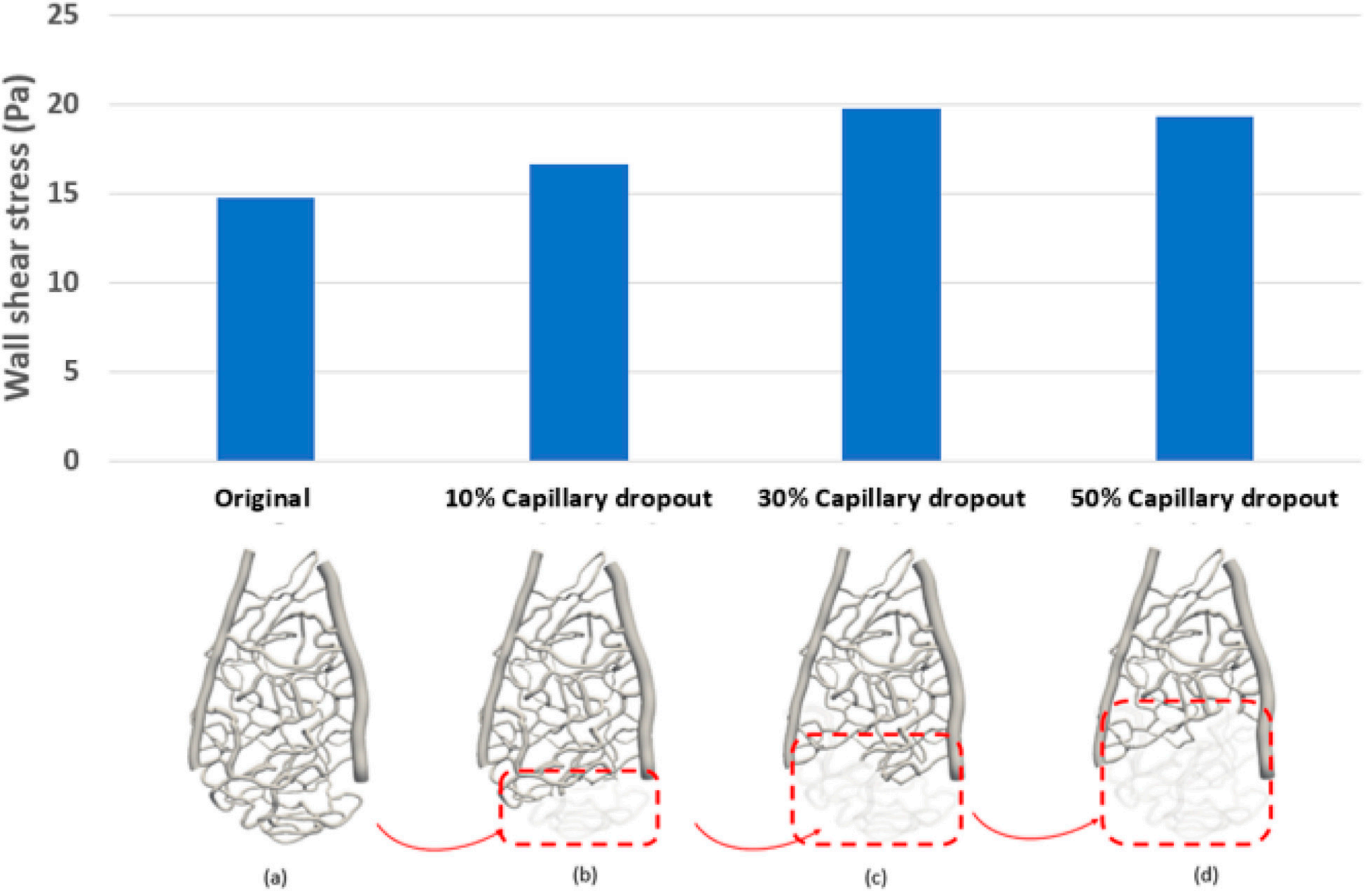
Average wall shear stress distribution under different percentages of capillary dropout: **(A)** original capillary network, **(B)** 10% capillary dropout, **(C)** 30% capillary dropout, and **(D)** 50% capillary dropout.

Capillary dropout was one of the most influential factors affecting wall shear stress, as shown in Figure 3. A 30% loss of the capillary network resulted in more than a 30% increase in wall shear stress and over a 20% increase in average velocity, as depicted in Figure 4.

### 3.3 Effects of venule pressure on wall shear stress distribution

**FIGURE 5:**
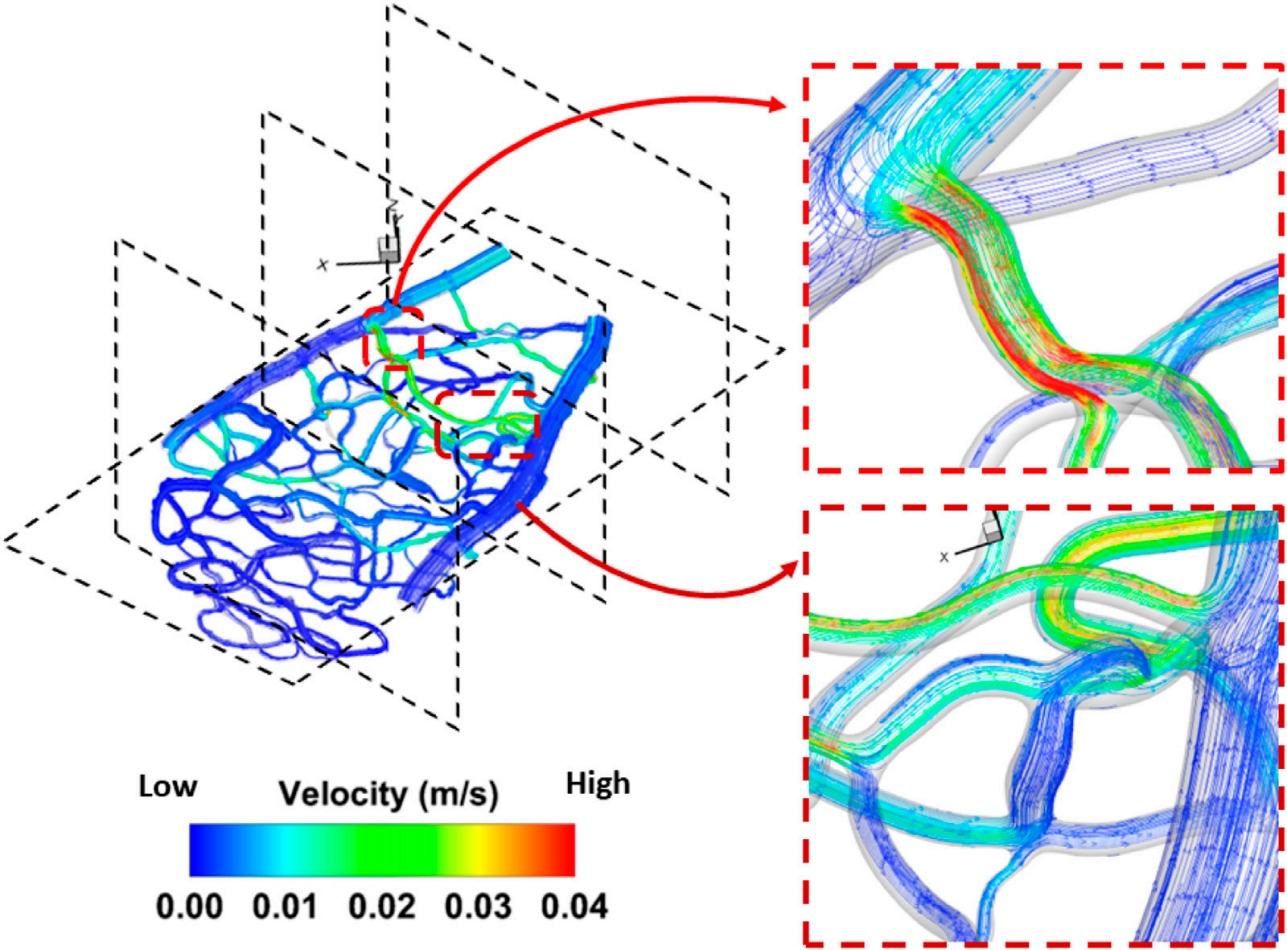
Detailed flow pattern in the rat 1 section 1. A disturbed flow region was found in the bifurcation region triggered by a change of diameter in the capillary network. The flow pattern for the remaining geometry is included in Supplementary Figure A2.

Venous pressure was the second most influential factor affecting both average pressure and average wall shear stress in the capillary network. In general, a 20 mmHg increase in pressure compared to the control case (100 mmHg) resulted in a 23% decrease in wall shear stress. Figure 5 shows both forward and backward streamline patterns, highlighting the velocity magnitude of the flow during the steady-state study. As the flow moves from the arterioles into the center of the capillary network, the streamlines become less uniform due to the transition from larger to smaller vessels, leading to the formation of vortices. In all cases, an obvious separation region is observed immediately after the tapered section. However, minimal separation occurs in the narrowing regions (vessels with smaller diameters) and gradually diminishes as the flow approaches the outlet of the capillary network further from the larger vessels (arterioles and venules). In addition, as the flow passes through the center of the capillary network and moves into the side vessel, low-velocity, irregular streamlines are observed beyond the central region. It is evident that the velocity magnitude increases in vessels as their diameter narrows. With greater narrowing of the capillary vessels, a high-velocity jet of blood flow becomes more pronounced, striking the inner vessel wall with increasing intensity. This leads to a noticeable increase in the velocity magnitude, indicating a significant shift in flow dynamics. In the region where the small capillaries transition into the venules, disordered streamlines are observed at both the entry point and beyond the vessel narrowing. A large-scale helical flow, characterized by the spiral motion of blood, develops in the venules, accompanied by the formation of a high-velocity jet, a concentrated stream of fluid, in areas of extreme narrowing where the vessel diameter significantly decreases.

**FIGURE 6:**
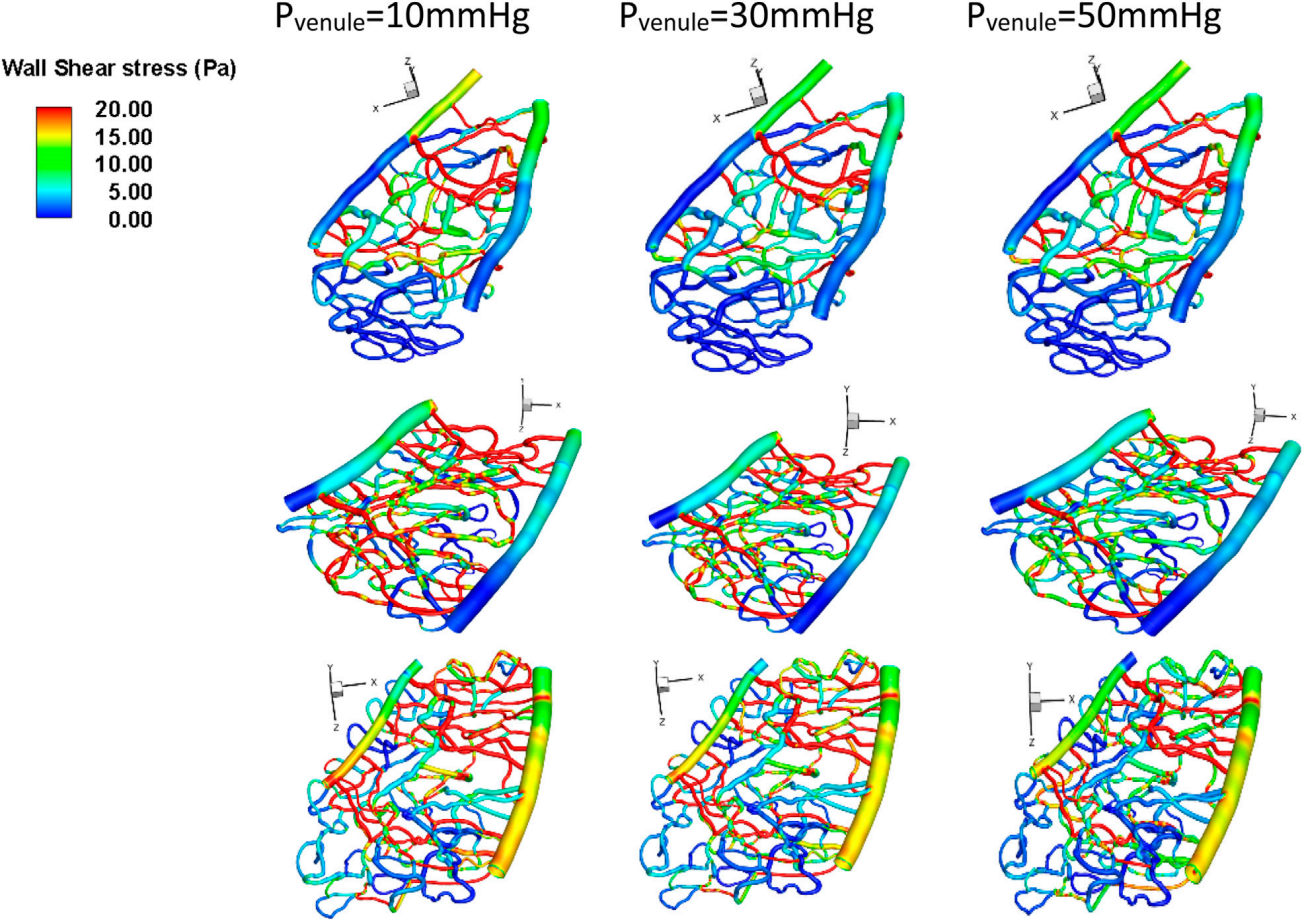
Wall shear stress under different venule pressure conditions for various geometries. Top row: Section 1, middle row: Section 2, bottom row: Section 3.

The average wall shear stress (Pa) is shown in Figure 6. Notably, the wall shear stress in section 1, which is farther away from the optic disc, was significantly higher than in sections 2 and 3, which are closer to the optic disc. Additionally, when there was more than 30% capillary dropout, the average wall shear stress was significantly higher (>3 Pa) than only a 10% capillary dropout, which results in only a 10% change in wall shear stress magnitude. The overall average wall shear stress across these three capillary sections was 14 ± 3.84 Pa. We also found that regions with high wall shear stress (>15 Pa) were primally located in the middle area between the arterioles and venules.

### 3.4 Effects of arteriole pressure on wall shear stress distribution

**FIGURE 7:**
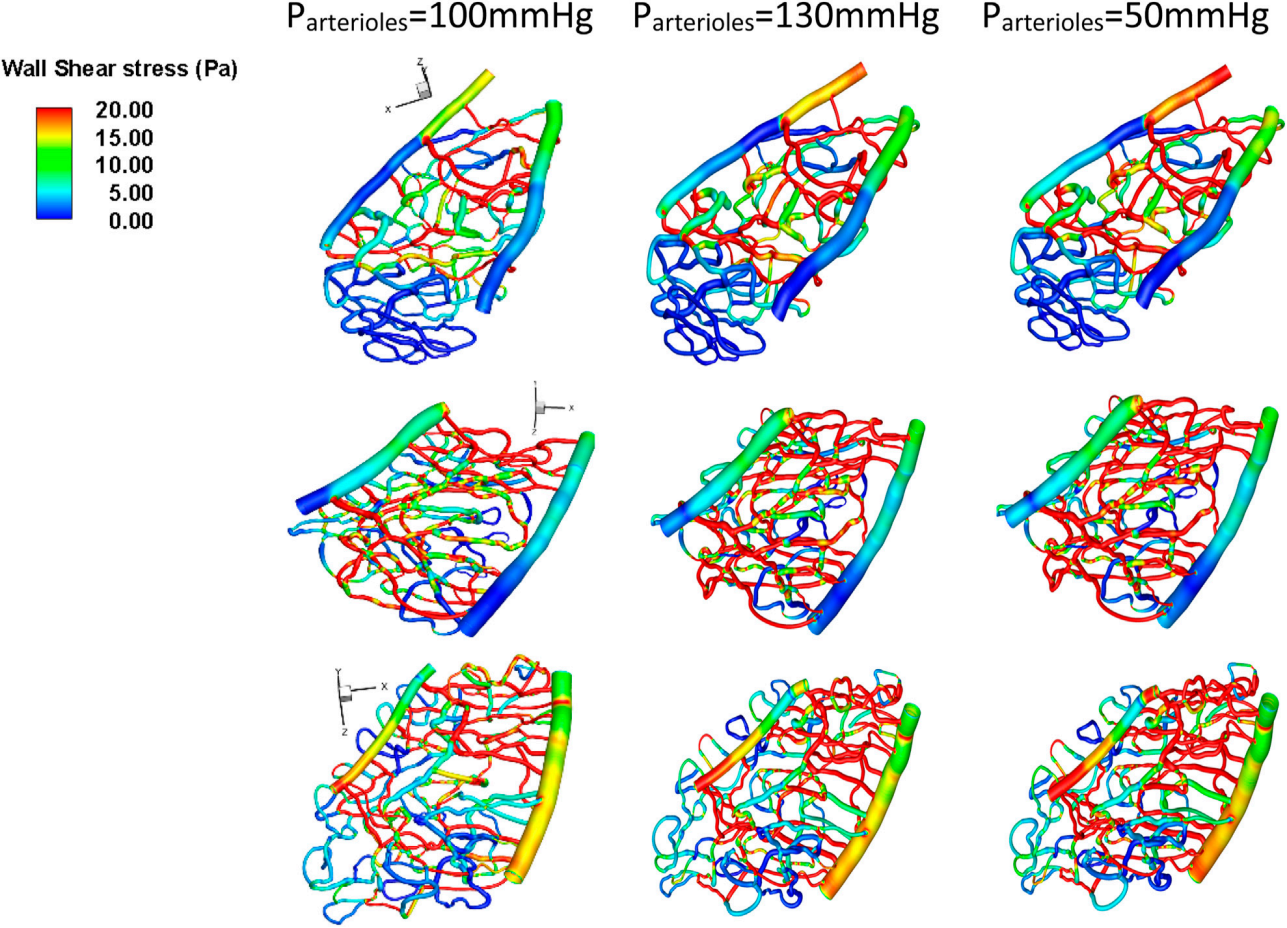
Wall shear stress under different arteriole pressure conditions for various geometries. Top row: Section 1, middle row: Section 2, bottom row: Section 3.

Arteriole pressure ranked last among all the parameters in terms of its influence on both average velocity and average wall shear stress in the capillary network. In general, a 30 mmHg increase in the arteriole pressure compared to control cases (100 mmHg) resulted in a 23% decrease in wall shear stress, as shown in Figure 7.

## 4 Discussion

Despite the common perception of glaucoma as a disease primarily influenced by intraocular pressure (IOP), evidence suggests that changes in venule hemodynamics can also impact its progression (Boyd et al., 2007). However, the precise relationship between changes in hemodynamic forces within the capillary network and the progression of ocular diseases requires more detailed investigation. This study examined changes in hemodynamic features (vascular pressure) and morphological alterations (the removal of partial capillary vasculature) that may predict the progression of ocular disease based on high-resolution confocal imaging. This approach allowed us to clearly define the wall boundaries of the rat capillary network and to include fine anatomic structures in our simulations, leading to a more realistic estimation of the WSS in the rat retina.

By using 3D confocal images of the rat retina to perform the simulation, we found that our segmented rat retinal capillary layer exhibited visible differences compared to those reported in the literature, which used a single 2D image from confocal microscopy (Bernabeu et al., 2014). Additionally, we found that the calculated WSS in the 3D rat retinal capillary network is comparable to the published literature (Bernabeu et al., 2014), with the highest value being 20 Pa. In the rat retinal capillary network, retinal capillaries exhibiting single-file flow were found to have small diameters, with a maximum lumen diameter of up to 7 µm (Guevara-Torres et al., 2015; Joseph et al., 2018). Omitting even small changes in the 3D capillary network can lead to substantial discrepancies in the calculated wall shear stress (WSS) compared to a 2D network. This is because the vertical component of blood flow in a 3D network significantly impacts WSS, which cannot be accurately captured by a 2D geometry constrained to a single plane. Our results indicate that the narrowing of retinal capillary tubes in the vertical direction is crucial for generating WSS in this network. Without 3D vessel reconstruction, the squeeze flow through the narrowing vessel in the vertical direction may not be accurately modeled, potentially leading to inaccuracies in simulating the flow dynamics. We believe that accurately capturing the boundaries of the vessels along the z-axis (vertical axis) in the rat retinal capillary network is essential for reliable WSS quantification. Although we did not directly vary the vertical blood vessels, the z-axis in our coordinate system corresponds to the anterior–posterior direction in physical space. Defining this coordinate system in our figures ensures clarity in the relationship between our model and the actual anatomical orientation. Furthermore, we demonstrate that a proper estimation of the 3D geometry is crucial for WSS estimation. The WSS in the second-order arterioles—defined as smaller branching vessels that arise from the first-order arterioles—was significantly lower in our study than that in the first-order arterioles. However, the WSS in the first-order venules was similar to that in the second-order venules, which aligns with the findings reported in the literature (Joseph et al., 2018).

Published literature shows that the flow in microvasculature is heterogeneous and exhibits significant fluctuations with a wide range of measured values (Schmid et al., 2017). In our study, the consistency of our results with the published measurements (Zhi et al., 2011) suggests that our assigned boundary conditions and numerical framework are appropriate. This alignment indicates that our methodology accurately captures the hemodynamic behavior observed in prior studies, supporting the validity of our approach.

The capillary networks we chose were significantly different in structure from each other. The change in diameter of arterioles and venules was found to significantly affect the hemodynamic forces in our study. Animals with smaller retinal arteriole diameters exhibited a higher change in shear rate than those with larger diameters (Leskova et al., 2020). However, in our current study, wall shear stress did not show significant differences under conditions of elevated venous pressure or across different vascular structures. The local hemodynamic changes included in the present study appear to differ from previous models, as most of the studies presented focused on a model reconstructed from the geometry derived from a single 2D image. Therefore, the flow entering the capillary network in both horizontal and vertical directions, as well as the direction of forward and backward flow, can change the magnitude of wall shear stress. This may help explain why some animals exhibit high wall shear stress, but not all experiments reach the same conclusion.

Pronounced changes in pressure are evident in arterioles, capillaries, and venules with IOP elevation, as noted in the literature (Leskova et al., 2020). Capillary perfusion pressure has been found to alter WSS across the vascular network, significantly affecting the hemodynamic pattern within the capillary network (Hossain et al., 2023). Wall shear stress is often associated with a change in endothelial cell behavior, which can moderate the adverse effects of high-pressure gradients. Change in WSS triggers a series of signaling pathways, resulting in the release of vasoconstrictors or vasodilators, such as nitric oxide (NO), that regulate the constriction of smooth muscle cells (SMCs) and ultimately lead to arterial wall disease (Brozovich et al., 2016; Freed and Gutterman, 2017). Through our simulations, we found that even without significantly altering the pressure gradient, changes in vascular structure can generate enough perturbation in velocities across the capillary networks to affect the magnitude of wall shear stress. As capillary density decreases, wall shear stress may initially drop significantly. However, once a certain threshold is reached—where only a few capillaries remain functional—the wall shear stress may stabilize or change at a slower rate despite further reductions in capillary density. This is because the remaining capillaries can still maintain a certain level of flow, albeit reduced. Additionally, low wall shear stress is closely correlated with vessel angiogenesis, which can lead to vessel remodeling (Ribatti and Crivellato, 2012; Freed and Gutterman, 2017; Roux et al., 2020).

Other evaluated anatomical features included total volume change and overall surface area; however, none of these features exhibited significant changes in hemodynamic forces with venous pressure below 50 mmHg. It was only when the venous pressure exceeded 50 mmHg that we observed a significant change in hemodynamic forces. Nowadays, clinicians mainly rely on elevated intraocular pressure (IOP) to predict the potential progression of ocular diseases and the risk of blindness. Our preliminary study shows that in addition to IOP, hemodynamic parameters such as venous pressure and mean ocular perfusion pressure should also be investigated when assessing the risk of ocular disease progression.

In the present study, we do not account for variations in vascular diameter resulting from fluid–structure interaction (FSI). Incorporating the influence of FSI remains a complex challenge that we plan to address in future research. However, according to the change, there is not much deformation of the capillary wall, and the stiffness of the capillary wall can be hard to measure. Furthermore, we do not take into account the pulsatility of the aortic pressure waveform, which will be taken into account in a future *in vivo* study.

## 5 Conclusion

Our simulations demonstrate that, in addition to the pressure gradient between arterioles and venules, changes in vascular structure can also affect the hemodynamic pattern of the capillary network. Changes in vascular structure were found to be highly correlated with variations in venule pressure. Although no significant differences in pressure were observed across different sections of the vascular network, elevated venule pressure and partial loss of vasculature may contribute to changes in wall shear stress, which could serve as a good indicator of vessel dilation. These findings are promising and suggest a potential role of hemodynamic forces in determining the risk of ocular disease progression following surgical treatment. Further studies in large animal models *in vivo* are warranted to validate these findings.

## Data Availability

The original contributions presented in the study are included in the article/Supplementary Material; further inquiries can be directed to the corresponding authors.
