# Supplementary material for: Evaluation of the effect of hemodynamic factors on retinal microcirculation by using 3D confocal image-based computational fluid dynamics
Source: Front Bioeng Biotechnol. 2024 Nov 27;12:1489172. doi: 10.3389/fbioe.2024.1489172 (PMC11631608; doi:10.3389/fbioe.2024.1489172)
Supplement: Supplementary file 1 [file DataSheet1.docx]

**Appendix A1**

| 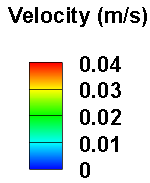 | 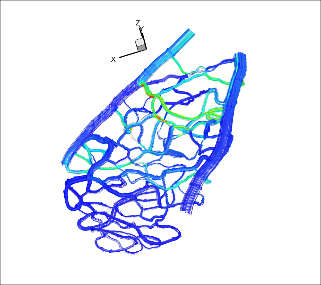 | 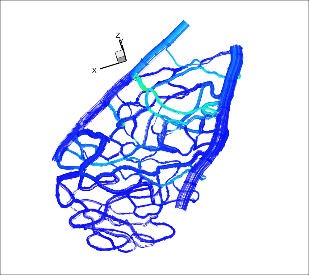 | 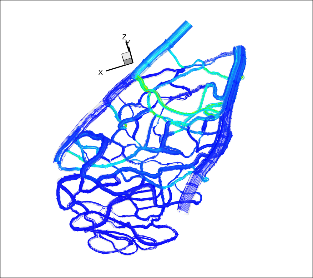 |
| --- | --- | --- | --- |
|  | 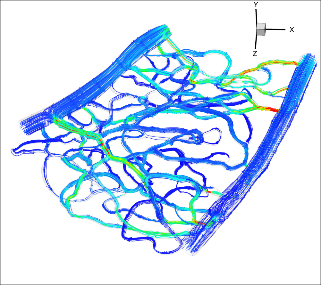 | 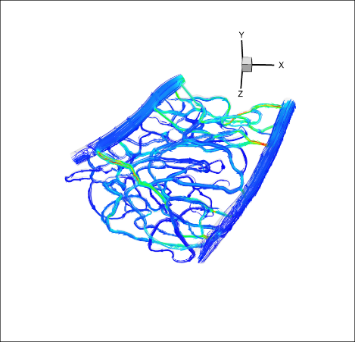 | 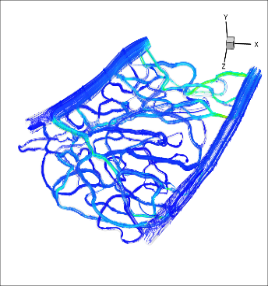 |
|  | 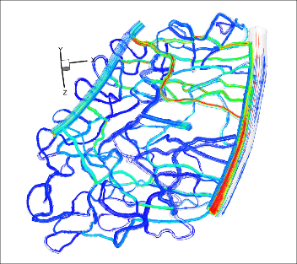 | 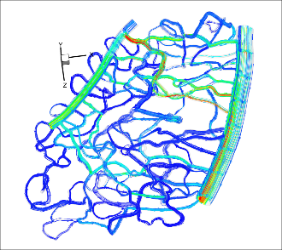 | 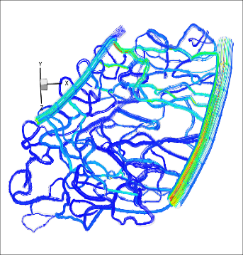 |

**Figure A1** Distribution of velocity in different geometries. Top row: Section 1, middle row: Section 2, bottom row: Section 3.

|  | P_venule_=10mmHg | P_venule_=30mmHg | P_venule_=50mmHg |
| --- | --- | --- | --- |
| 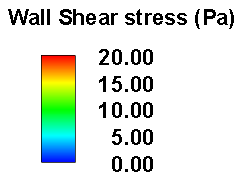 | 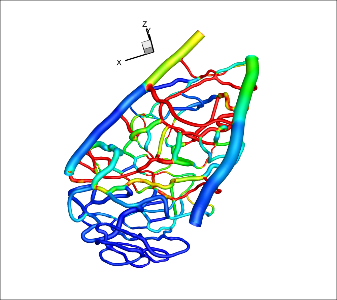 | 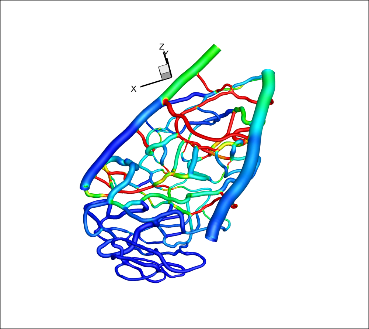 | 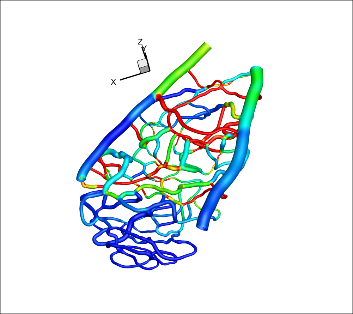 |
|  | 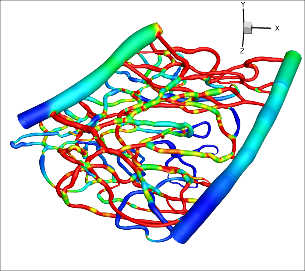 | 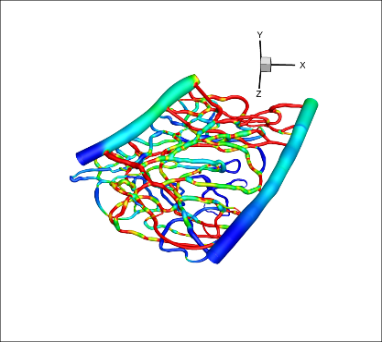 | 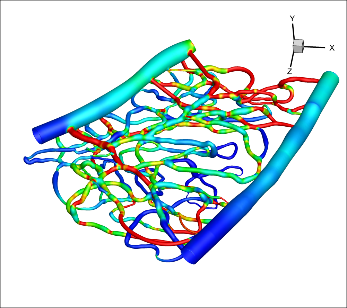 |
|  | 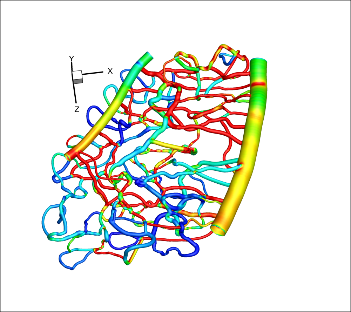 | 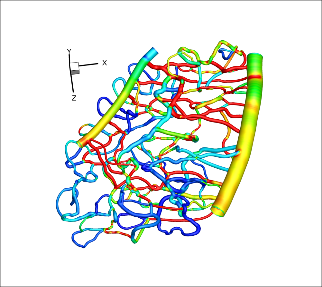 | 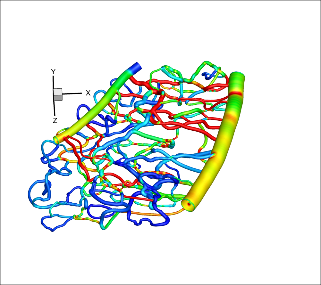 |

**Figure A2** Wall shear stress under different venule pressure conditions for various geometries. Top row: Section 1, middle row: Section 2, bottom row: Section 3.

**Appendix A2**

Dice coefficient (also known as the Sørensen-Dice index) is a statistical measure used to gauge the similarity between two sets, commonly employed in image analysis, particularly in the fields of medical imaging and computer vision.

**Definition**

The Dice coefficient is defined as

$$Dice\left( A, B \right)=\frac{2| A\cap B|}{\left| A \right|+|B|}$$

where

*A* and *B* are two sets (in the content of images, these could be sets of pixels or regions).

|*A*| is the number of elements in set A

|*B*| is the number of elements in set *B*

$|A\cap B|$Is the number of elements common to both sets.

**Interpretation**

- The Dice coefficient ranges from 0 to 1:
  - A value of **1** indicates perfect overlap between the two sets (i.e., they are identical).
  - A value of **0** indicates no overlap.

In image segmentation tasks, the Dice coefficient is often used to evaluate the performance of algorithms by comparing the predicted segmentation against a ground truth segmentation. It helps quantify how well the algorithm captures the intended regions of interest in the image.

**TABLE A1** Mesh sensitivity test for the original model.

| Mesh type | Number of elements | averaged WSS (Pa) |
| --- | --- | --- |
| Coarse mesh | 7944423 | 14.6314 |
| Fine mesh | 8472809 | 14.7319 |
| Finer mesh | 10484549 | 14.7701 |
